# Supplementary material for: An open-source framework for large-scale, flexible evaluation of biomedical text mining systems
Source: J Biomed Discov Collab. 2008 Jan 29;3:1. doi: 10.1186/1747-5333-3-1 (PMC2276192; doi:10.1186/1747-5333-3-1)
Supplement: Additional file 1 — Evaluation platform source code. The additional file contains the software evaluation framework discussed in this paper. The most current release can be downloaded from SourceForge.net [1]. See the accompanying README file for instructions on installation and use. Also included in the distribution is a collection of UIMA wrappers for some commonly used BioNLP tools and annotated corpora. [file 1747-5333-3-1-S1.zip › bionlp_uima_repository-1.0.1/docs/api/edu/uchsc/ccp/uima/mention/package-summary.html]

edu.uchsc.ccp.uima.mention (Center for Computational Pharmacology Util/UIMA API)


|  |  |  |
| --- | --- | --- |
| **Overview** \| **Package** \| Class \| **Tree** \| **Deprecated** \| **Index** \| **Help** | |  |
| **PREV PACKAGE**   **NEXT PACKAGE** | **FRAMES**    **NO FRAMES**     **All Classes** |  |


---

## Package edu.uchsc.ccp.uima.mention

| **Class Summary** | |
| --- | --- |
| **CCPClassMention** | The CCP ClassMention is the root of a flexible class structure that can be used to store virtually any frame-based representation of a particular class. |
| **CCPClassMention\_Type** | The CCP ClassMention is the root of a flexible class structure that can be used to store virtually any frame-based representation of a particular class. |
| **CCPComplexSlotMention** | A slot mention is deemed "complex" when its slot filler is a class mention as opposed to a String (See non-complex slot mention for String fillers). |
| **CCPComplexSlotMention\_Type** | A slot mention is deemed "complex" when its slot filler is a class mention as opposed to a String (See non-complex slot mention for String fillers). |
| **CCPMention** | The superclass for all CCP Mentions (class mention, complex slot mention, and non-complex slot mention) Updated by JCasGen Tue Aug 28 16:44:10 MDT 2007 XML source: /home/williamb/eclipse/RELEASED\_SOURCE\_CODE/descriptors/typeSystem/CCPTypeSystem.xml |
| **CCPMention\_Type** | The superclass for all CCP Mentions (class mention, complex slot mention, and non-complex slot mention) Updated by JCasGen Tue Aug 28 16:44:10 MDT 2007 |
| **CCPNonComplexSlotMention** | The non-complex slot mention has slot values which are constrained to be Strings. |
| **CCPNonComplexSlotMention\_Type** | The non-complex slot mention has slot values which are constrained to be Strings. |
| **CCPSlotMention** | The superclass for all slot mentions (complex and non-complex). |
| **CCPSlotMention\_Type** | The superclass for all slot mentions (complex and non-complex). |

---


|  |  |  |
| --- | --- | --- |
| **Overview** \| **Package** \| Class \| **Tree** \| **Deprecated** \| **Index** \| **Help** | |  |
| **PREV PACKAGE**   **NEXT PACKAGE** | **FRAMES**    **NO FRAMES**     **All Classes** |  |


---
